# Increased Serum Expression of Inflammatory Cytokines may Serve as Potential Diagnostic Biomarker for Bilirubin Encephalopathy

**DOI:** 10.6061/clinics/2020/e1868

**Published:** 2020-11-25

**Authors:** Hanzhou Guan, Chenghu Wang, Xinhua Zhang

**Affiliations:** Department of Neonatology, Shanxi Provincial Children’s Hospital, Taiyuan, China

**Keywords:** BE, IL-1β, TGF-β, Biomarker

## Abstract

**OBJECTIVES:**

The present study was designed to explore the roles of inflammatory cytokines interleukin-1β (IL-1β) and Tumor growth factor-β (TGF-β) in the diagnosis and treatment of neonate bilirubin encephalopathy (BE).

**METHODS:**

A total of 128 BE neonates and 128 normal neonates were included. The serum samples of the BE children and controls were collected, and the levels of IL-1β and TGF-β were examined. Moreover, the correlation between the level of bilirubin and serum expression of IL-1β or TGF-β in BE patients was analyzed. Finally, receiver operating characteristic (ROC) curves were generated to determine the diagnostic value of the cytokines.

**RESULTS:**

IL-1β and TGF-β levels were higher in the serum of BE patients than those in non-BE patients, and the expression of either IL-1β or TGF-β showed a strong positive correlation with the serum expression of bilirubin in BE patients. Moreover, the results of ROC analysis showed that either IL-1β or TGF-β could distinguish BE patients from healthy controls.

**CONCLUSION:**

IL-1β and TGF-β levels were upregulated in BE and might function as potential biomarkers or therapeutic targets for BE patients.

## INTRODUCTION

In the field of neonate diseases, some infants suffer from bilirubin encephalopathy due to the extremely high serum bilirubin levels and the development of bilirubin-induced neurologic injury (1-3). Bilirubin encephalopathy (BE, also known as kernicterus) can be classified as acute or chronic BE based on the duration of the disease (1). Although considerable efforts have been made to explore the pathogenesis of the disease and to constantly improve the medical techniques, bilirubin encephalopathy continues to occur frequently. The prediction and diagnosis of bilirubin encephalopathy are dependent on the assessment of clinical symptoms, total serum bilirubin level, the ratio of serum bilirubin/albumin, and finally, on the results of magnetic resonance imaging (MRI) (4-6). In China, some researchers have used neonatal behavioral neurological assessment to assess clinical symptoms, but it is complex and less specific in predicting bilirubin encephalopathy.

Inflammation has been considered an important event during the occurrence and progression of BE (7,8). The activation of nuclear factor-kappa B (NF-κB) signaling, which is known as the key signaling pathway during the process of inflammation, as well as several cytokines, were found to be abnormally expressed in BE and associated with the development of the disease (7,9). Therefore, in the current study, the roles of pro-inflammatory cytokines, including interleukin-1β (IL-1β) and tumor growth factor-β (TGF-β), in BE have been investigated. We designed the present study to investigate the potential clinical significance of measuring the expression level of IL-1β or TGF-β in the serum of BE newborns as a biomarker for the diagnosis and treatment of BE.

## MATERIAL AND METHODS

### Patients

The present work included 128 BE newborns and 128 healthy controls from the Department of Neonatology, Shanxi Provincial Children’s Hospital between May 2017 and July 2019. The criteria used for inclusion were: (1) neonates who were diagnosed with hyperbilirubinemia (defined as a total serum bilirubinemia (TSB) level >221 μmol/L); (2) 37 gestational age: 37-42 weeks; (3) hyperbilirubinemia that was diagnosed within 7 days. The criteria used for exclusion were: neonates who died within 12 h after birth, neonates with incomplete data and information, those with intracranial infection, congenital malformation (chromosome abnormality), and family history of deafness. Severe hyperbilirubinemia: total serum bilirubinemia (TSB) >342 μmol/L; very severe hyperbilirubinemia >427 μmol/L; bilirubin encephalopathy: apart from TSB>342 (1). There were typical symptoms of nervous system disorders, such as lethargy, convulsion, dystonia, and asthenia. Head magnetic resonance imaging (MRI) showed characteristic changes in T1-weighted (T1W) and T2-weighted (T2W) images of the bilateral globus pallidus with high signal symmetry. Brain stem auditory evoked potential (BAEP) suggests a high frequency of hearing loss, which is diagnosed according to severe hyperbilirubinemia and clinical manifestations. This study was approved by the Shanxi Provincial Children's Hospital Ethics Committee, and all candidates signed written informed consent forms.

### General information

General information included gestational age (week), admission age (h), sex (male/female), birth weight (g), maternal gestational diabetes, production type (natural childbirth *versus* caesarean), meconium delay, and birth asphyxia. Serum levels of bilirubin were detected using an automatic biochemical analyzer (Bayer Diagnostics, Berkshire, UK).

### Enzyme-linked immunosorbent assay

The expression levels of IL-1β and TGF-β in the serum samples of BE patients and healthy subjects were detected using enzyme-linked immunosorbent assay (ELISA) by using kits purchased from Beyotime (Haimen, China). The procedures in the instructions provided by the manufacturer were strictly followed.

### Statistical analysis

Data of the present work are expressed as mean ± standard deviation (SD). Comparisons between the BE and non-BE groups were detected using the Student’s *t*-test and GraphPad Prism v7.0 software (GraphPad, CA, USA). Receiver operating characteristic (ROC) curve analysis has been used to predict the potential diagnostic value of IL-1β and TGF-β for BE. Pearson’s correlation coefficient was used for the analysis of correlation between variables. Significance was considered when the p-value was less than 0.05.

## RESULTS

### General information of the candidates

All the participants (60.2%) were boys and 39.8% of the selected neonates were girls. The mean gestational age was 38.1 weeks. The mean birth weight was 3,232.5 g. Among the mothers of the neonates, 21.4% had gestational diabetes.

### Pro-inflammatory cytokines are over-expressed in the serum of BE patients

**Figure 1:**
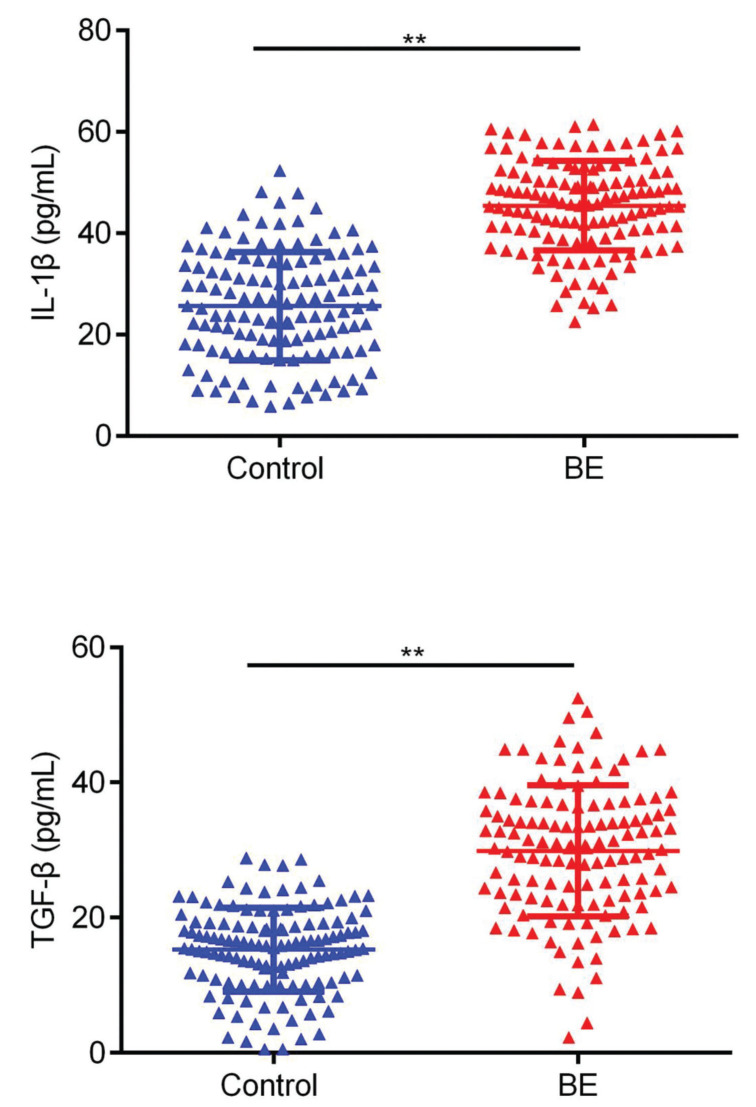
Comparison of the serum expression of IL-1β, as well as TGF-β, in BE patients and healthy controls. **p<0.01.

First, we evaluated the expression levels of IL-1β and TGF-β (commonly categorized as pro-inflammatory cytokines) in BE patients and healthy volunteers using ELISA. As revealed by the results in Figure 1, the levels of IL-1β and TGF-β were markedly upregulated in the serum of patients with BE in comparison with those of healthy subjects (Figure 1, *p*<0.01).

### Correlation between the expression of bilirubin and the expression of IL-1β, as well as TGF-β, in serum samples of patients with BE

**Figure 2:**
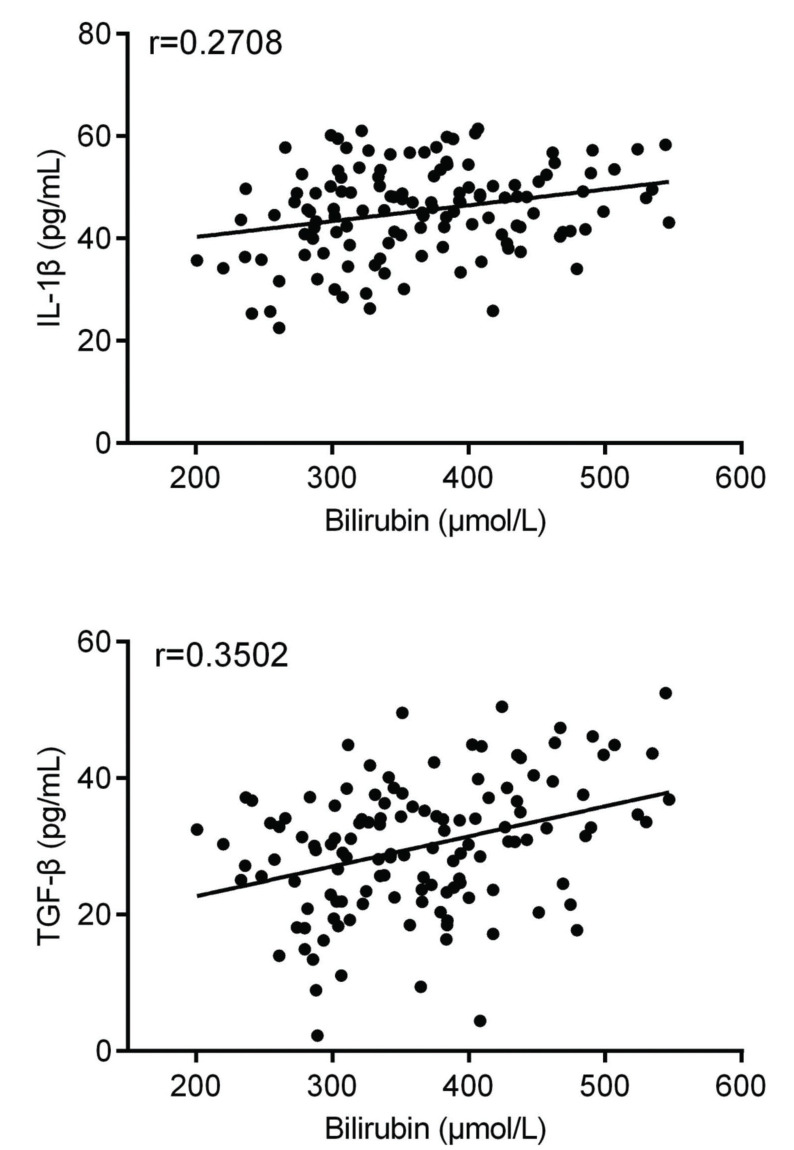
Correlation between the serum levels of bilirubin and IL-1β, as well as TGF-β, in BE patients.

**Figure 3:**
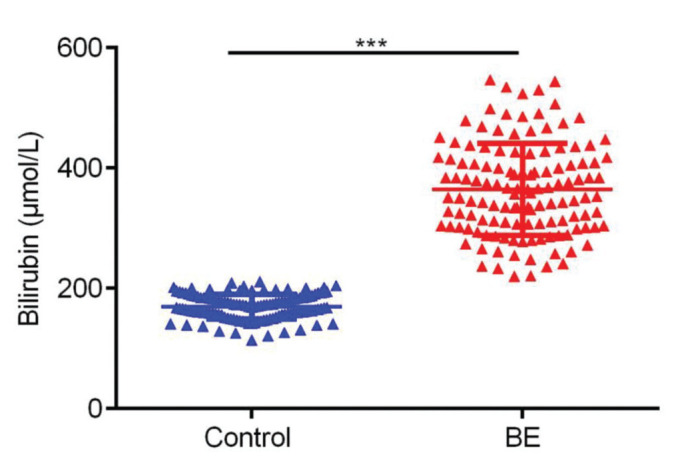
Comparison between the serum expression of bilirubin in BE patients and healthy controls. **p<0.01.

As shown in Figure 2, bilirubin levels were significantly increased in BE patients compared to those in controls (*p*<0.001). Furthermore, we performed Pearson’s correlation analysis to analyze the relationship between the expression of bilirubin and the expression of IL-1β and TGF-β in BE neonates; Figure 3 shows the results of this analysis. We found that the expression levels of bilirubin were positively correlated with the level of IL-1β in serum samples (r=0.2708, *p*=0.002) and TGF-β levels in serum samples (r=0.3502, *p*<0.001) in patients with BE.

### Potential diagnostic value for detecting the expression of IL-1β, as well as TGF-β, in the serum of BE patients

**Figure 4:**
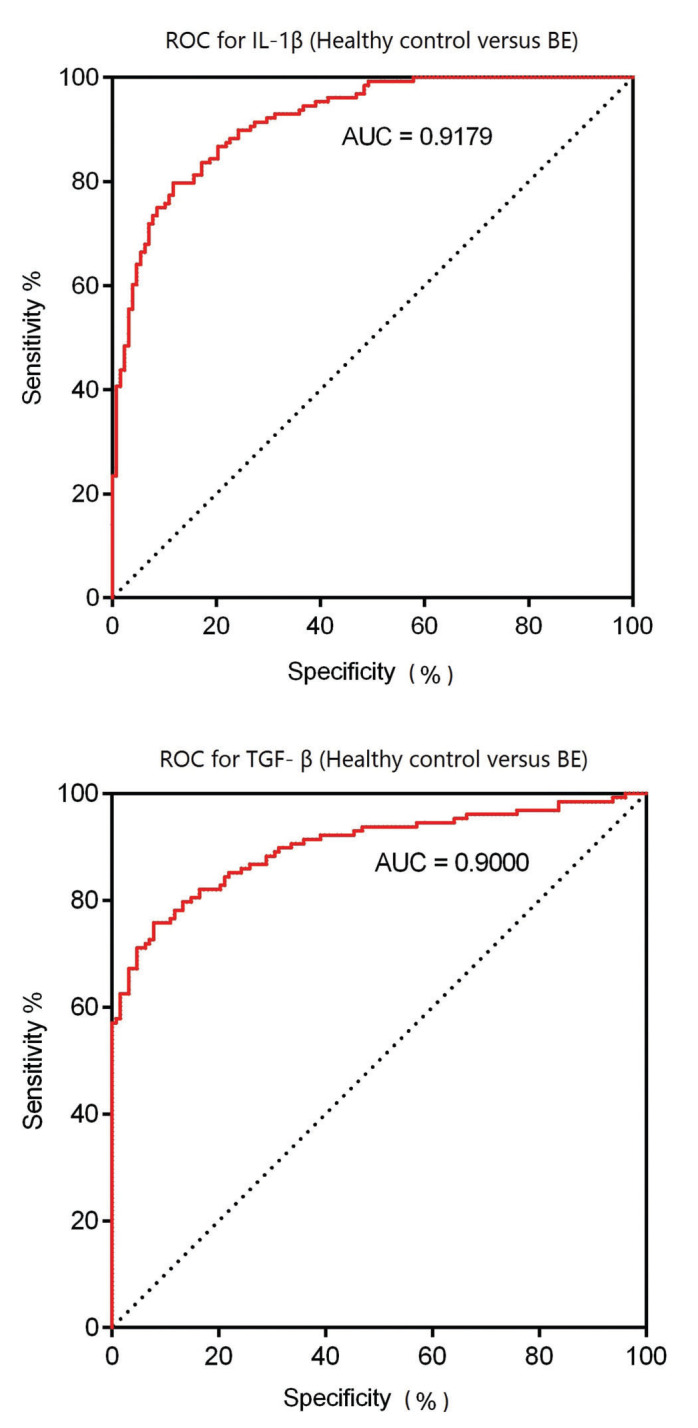
Results of ROC analysis.

Finally, the potential diagnostic values of IL-1β and TGF-β for BE were evaluated using receiver-operating characteristic curves (ROC). As shown in Figure 4, the area under the curve (AUC) value for the expression level of IL-1β to distinguish BE patients from healthy subjects was 0.9179 (95% confidence interval [CI], 0.8858 to 0.9500; cut-off value, 37.97). Moreover, the AUC value for TGF-β was 0.9000 (95% CI, 0.8608-0.9391; cut-off value, 21.82). The above-mentioned results indicate that IL-1β and TGF-β may serve as sensitive biomarkers for the early diagnosis of BE.

## DISCUSSION

In the current study, the expression levels of IL-1β and TGF-β in the serum of BE neonates and their clinical significance have been explored. It was observed that the levels of IL-1β and TGF-β were dramatically increased in BE patients, and the levels of these inflammatory cytokines were correlated with disease severity. Moreover, both IL-1β and TGF-β could function as diagnostic biomarkers for BE.

Several previous studies have elucidated the role of inflammation in the development of BE. For example, a previous study suggested that bilirubin could induce the immune response of astrocytes and microglia, including an increased expression of inflammatory cytokines, such as IL-1β and IL-6 (10). Moreover, it has been reported that inhibition of NF-κB inflammatory signaling exerts neuroprotective functions in BE rat models (9). IL-1β (11,12) and TGF-β (13-15) are two common cytokines secreted by immune cells; they were found to be upregulated in many immune diseases (11,15); however, their expression patterns, as well as their clinical significance in BE, have not been discussed thus far. In the current study, we found that both IL-1β and TGF-β expression levels were markedly increased in the serum of BE patients compared with those of healthy controls. Moreover, we also found that among BE patients, the serum levels of IL-1β and TGF-β showed a strong positive correlation with the levels of bilirubin. As the upregulated level of bilirubin was considered as the main cause of the disease (8,16), these results suggested that the levels of these inflammatory cytokines were correlated with the severity of BE. Taken together, our data suggested that IL-1β and TGF-β expression levels were upregulated in BE and that they were associated with the occurrence and development of the disease.

Moreover, the early diagnosis of BE is important for physicians to make effective decisions before appearance of the symptoms and to subsequently reduce the morbidity and mortality of the disease (4). Several other studies have discussed the potential diagnostic value of some biomarkers for the early diagnosis of BE (1,6,16,17). In our study, we performed ROC analysis, and the results indicated that either IL-1β or TGF-β could distinguish BE patients from healthy controls with a high specificity and sensitivity. Therefore, our data suggest the diagnostic value of the serum levels of IL-1β and TGF-β for clinical application. However, the results require validation using more clinical samples from different countries and from individuals of different ethnicities/races.

The current study has some limitations. First, the results of the current work require further investigation with a larger cohort. Moreover, the current work was based on the Chinese Han population, and the results need to be validated for samples obtained from people of other races.

## CONCLUSION

In summary, IL-1β and TGF-β were dramatically upregulated in the serum of BE patients, and both these inflammatory cytokines could be used for the early diagnosis of BE. The current study provides novel evidence for the potential clinical application for detection of the expression of either IL-1β or TGF-β for the diagnosis and treatment of BE.

## AUTHOR CONTRIBUTIONS

Hanzhou Guan contributed in data curation (lead), formal analysis (lead), investigation (lead), and methodology (lead). Chenghu Wang contributed in data curation (Supporting), formal analysis (Supporting), Investigation (Supporting), methodology (Equal), writing-review, and editing (Supporting). Xinhua Zhang contributed in conceptualization (lead), supervision (lead), writing original draft (lead), writing-review, and editing (lead).
